# Schizophrenia: A Survey of Artificial Intelligence Techniques Applied to Detection and Classification

**DOI:** 10.3390/ijerph18116099

**Published:** 2021-06-05

**Authors:** Joel Weijia Lai, Candice Ke En Ang, U. Rajendra Acharya, Kang Hao Cheong

**Affiliations:** 1 Science, Mathematics and Technology, Singapore University of Technology and Design, 8 Somapah Road, Singapore 487372, Singapore; joel_lai@mymail.sutd.edu.sg (J.W.L.); candiceangk@gmail.com (C.K.E.A.); 2 MOH Holdings Pte Ltd, 1 Maritime Square, Singapore 099253, Singapore; 3 Department of Electronics and Computer Engineering, Ngee Ann Polytechnic, Clementi 599489, Singapore; aru@np.edu.sg; 4 Department of Biomedical Engineering, School of Science and Technology, Singapore University of Social Sciences, Clementi 599491, Singapore; 5 Department of Biomedical Informatics and Medical Engineering, Asia University, Taichung 41354, Taiwan

**Keywords:** artificial intelligence, machine Learning, mental health, schizophrenia

## Abstract

Artificial Intelligence in healthcare employs machine learning algorithms to emulate human cognition in the analysis of complicated or large sets of data. Specifically, artificial intelligence taps on the ability of computer algorithms and software with allowable thresholds to make deterministic approximate conclusions. In comparison to traditional technologies in healthcare, artificial intelligence enhances the process of data analysis without the need for human input, producing nearly equally reliable, well defined output. Schizophrenia is a chronic mental health condition that affects millions worldwide, with impairment in thinking and behaviour that may be significantly disabling to daily living. Multiple artificial intelligence and machine learning algorithms have been utilized to analyze the different components of schizophrenia, such as in prediction of disease, and assessment of current prevention methods. These are carried out in hope of assisting with diagnosis and provision of viable options for individuals affected. In this paper, we review the progress of the use of artificial intelligence in schizophrenia.

## 1. Introduction

**Figure 1:**
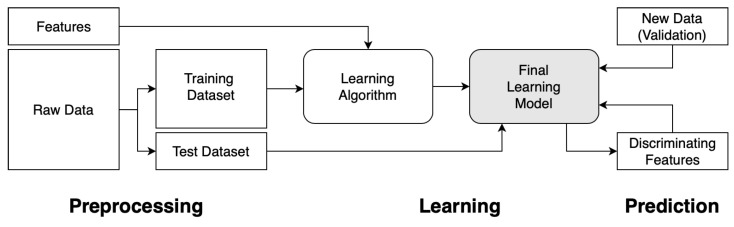
Flowchart to demonstrate the general framework of the process of training a machine learning algorithm.

Machine learning (ML) is the process of automating the tracking of changes in data patterns through a trained learning algorithm. Data is key in training of good learning models as it generates patterns for development of learning algorithms, in which future predictions are based upon. The unique features of each dataset form the discriminating factors for patterns generated, and hence the learning algorithm. Data can be split into a training set and a test set, to be used for evaluation. A ML algorithm is first selected and trained with the data from the training set with certain features collected. Features that prove not to provide discrimination are then removed as it can severely slow down training time or return false results. This process is then repeated and optimized to fine tune the learning model for achieving higher accuracies in prediction. It is then eventually applied to the test set or with new data for validation of the final learning model. This is the ML process. The flow of the process is captured in Figure 1.

Artificial intelligence (AI) and ML in the medical field has been advancing quickly since the advent of modern computers. With advances in computational power and the increased complexity of medicine, both AI and medicine has crossed paths and collaborations between both communities have increased with uncharted potential [1,2]. Advances in AI and ML is transforming our ability to analyze and process large amounts of data and to predict outcomes in biomedical research and healthcare delivery. AI and ML have been well explored for creating predictive models and have been used extensively in a variety of medical and healthcare purposes [3,4]. It can also transform the way that clinical decisions and clinical diagnosis are being made [5,6]. Examples include the classification and extraction of medical data [7,8], real-time analysis of medical scans [9], potential use of diagnosing medical conditions [10], and automate medical processes such as detection and classification [11]. Of focus in this review is the classification and diagnosis of mental health patients. Increasingly, researchers from ML and medical fields have sought to better classify and diagnose mental health cases thereby enabling a more accurate diagnosis and classification of mental health [12,13,14] to provide patients with personalized treatment programs to improve their recovery [15,16]. For these reasons, this course of research is increasingly deserving of attention and the collaboration of these two fields will continue to push the frontiers of learning.

Schizophrenia (*SZ*) is a severe chronic mental health condition that affects millions worldwide and associated with significant impairment of quality of life. At present, it is diagnosed clinically by fulfilling a criteria of phenotypical features over a temporal distribution as stated by either the Diagnostic and Statistical Manual of Mental Disorders, 5th edition (DSM-V) or the International Classification of Diseases 11th Revision (ICD-11) [17]. While it is not as common as other mental health disorders such as depression or anxiety, the symptoms of *SZ* are often disabling. People with *SZ* may seem like they have lost touch with reality [18,19]. Symptoms of *SZ* usually start at early ages of 16 to 30. The symptoms of *SZ* can be classified into three categories, namely positive, negative or cognitive symptoms [20,21]. Clinical assessments are performed based on these observed symptoms and corroborative reports [22]. Symptoms associated with *SZ* occur along a continuum and must be of considerable severity and impairment before a diagnosis is made [23].

*SZ* is characterized by hallucinations, delusions, disorganized speech, and other symptoms that cause social or occupational dysfunction such as impairments in cognition, attention and memory. It can only be diagnosed after exclusion of organic causes such as dementia or delirium that can manifest similarly. Treatment of *SZ* is generally classified under two broad categories—non-pharmacological and pharmacological. Non-pharmacological interventions such as cognitive behavioural therapy aim to help patients cope with their symptoms and achieve an acceptable level of psychosocial functioning in society. Pharmacological treatment remains the mainstay of therapy, based upon neurobiological theories of re-uptake and release of neurotransmitters such as glutamate, gamma aminobutyric acid, acetylcholine, and serotonin. More recently, methods such as electroconvulsive therapy have proven to be of benefit in the treatment of *SZ*. However, the treatment of *SZ* [24] is beyond the scope of the current review.

With technological advances, there are increasing efforts to “operationalize” and “objectify” the detection of *SZ*, with AI and ML techniques. Large amounts of data, ranging from investigations derived from magnetic resonance imaging (MRI) scans, positron emission tomography (PET) scans and electroencephalography (EEG) and subjective interpretations of patient’s posture, facial expression, word choices, attitude and behaviour, have been analyzed in attempt to define *SZ*. However, there have been few attempts to organize these studies in a systematic manner by presenting the number of subjects, AI and ML technique used, and prediction accuracy. In this review, we will synthesize the work presented by various research groups that employ the use of artificial intelligence and machine learning in classifying and detecting, and report their prediction accuracy.

The rest of the article is organized as such: Section 2 describes our methodology in curating existing literature, and the process of choosing which articles are suitable. In Section 3, we report on different machine learning techniques used for various input data types, such as MRI scans, the size of their samples and their classification accuracy. We provide perspective on the potential outlook on how to employ machine learning as a means to measure the effectiveness of furthering *SZ* research in Section 4, before concluding in Section 5.

## 2. Methodology

**Figure 2:**
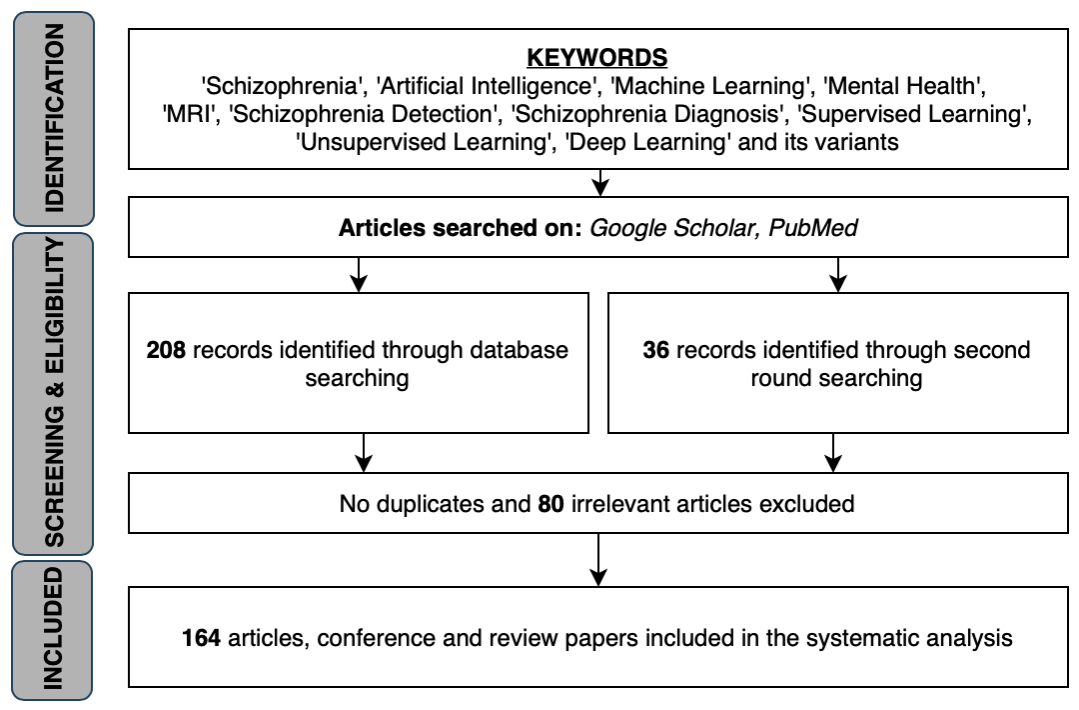
Procedural flow diagram choosing suitable literature.

In this systematic review, we did a search on articles, conference and review papers using key words such as ‘Schizophrenia’, ‘Artificial Intelligence’, ‘Machine Learning’, ‘Deep Learning’, ‘Mental Health’, ‘Detection’, ‘Diagnosis’ and its variants. The resulting literature were screened for relevance before chosen to be included in this review. A procedural flow diagram is included in Figure 2 to show the process for which suitable literature were chosen. The selected papers range from the Year 1999 to 2020. There has not been any work carried out thus far to consolidate key papers that have tapped on the technological advances in AI and ML with regards to *SZ*. As such, our paper will be the first of its kind to consolidate existing papers by presenting their study sample size, classification accuracies and the method used for classification.

## 3. Survey of AI Methods for Classification and Detection of Schizophrenia

AI techniques have been used in the detection of *SZ* via different means. The bulk of attempts to detect *SZ* stems from various types of MRI scans. Other techniques of detection using AI include PET scans, EEG and other techniques involving prediction through psycho-physio abilities and by gene and protein classification.

### 3.1. Classification and Detection of SZ by MRI

Magnetic resonance imaging is a medical imaging technique used in radiology to form images depicting anatomy. With various sequences, MRI may provide insight of physiological processes of the body. Scanned images of the brain were taken from both patients diagnosed with *SZ* and healthy controls [25]. These images were compared to detect *SZ* using various means of AI and ML tools. A typical MRI scan can allow medical professionals to diagnose the onset of *SZ*.

#### 3.1.1. Structural MRI

Structural MRI (sMRI) is the study of the structure of different parts of the brain and making predictions by comparing the MRI scans of patients and control subjects. By comparing the scans, ML algorithms can be trained to classify patients with and without *SZ*. Leonard et al. [26] was one of the first to use discriminant function analysis (DFA) to correctly classify the subjects (77% accuracy) from the structural brain scans. The bulk of the work in other sMRI techniques focus on analyzing and comparing Grey Matter (GM) and White Matter (WM), and their corresponding size or density. Other groups used DFA and its variants to classify and detect patients with *SZ* by considering other Region-of-Interest (ROI) in the brain and were able to achieve similar or better prediction rates by performing DFA on sMRI scans. Through the various studies, we have noticed that researchers tend to make the same conclusion—the risk of *SZ* may depend on the total amount of neural deviance rather than on anomalies in a single structure or circuit.

Another popular method used in classifying *SZ* is the use of support vector machine (SVM) classifiers, including non-linear SVM and its variants. SVM forms the majority of the analysis from detection using sMRI images. Customary in most predictive analysis, the SVM models were constructed from one set of subjects (training set) and the model was then applied to a different set of subjects (test set) to cross-validate the model. Many groups also used SVM to compare at-risk mental state (ARMS) *SZ* individuals with healthy controls (HC). In particular, in the work of Koutsouleris et al. [27], non-linear SVM with multivarite neuroanatomical pattern classification was performed on the sMRI data of individuals with ARMS (early and late) and HC. The accuracy of the method was then evaluated by categorizing the baseline imaging data of individuals with transition to psychosis as compared to those without transition and HC after 4 years of clinical follow-up. The 3-group, cross-validated classification accuracies of the first analysis were 86% in discriminating HC, 91% in discriminating early ARMS, and 86% in discriminating late ARMS. The accuracies in the second analysis were 90% in discriminating HC, 88% in discriminating individuals with transition, and 86% in discriminating individuals without transition. Independent HC were correctly classified in 96% (first analysis) and 93% (second analysis) of cases. Notably, there were several studies that point to better prediction accuracies when combining multiple features than simply employing single-modal features in SVM [28,29,30].

Other ML methods notably include the regression model used by Csernansky et al. [31] to predict *SZ* among subjects who were similar in age, gender and parental socioeconomic status, with 75% prediction rate. However, it was unable to predict the severity of the condition using the same model. Other notable methods employed include the high-dimensional non-linear pattern classification used by Davatzikos et al. [32] to quantify the degree of separation between patients and control, achieving 81.1% mean classification accuracy. An overview of the work, sample size and accuracy from utilizing machine learning techniques on structural magnetic resonance imaging data is compiled in Table 1.

#### 3.1.2. Functional MRI

Functional MRI (fMRI) scans display changes in blood oxygen level concentration as a consequence of task-induced or spontaneous modulation of neural metabolism. The strength of fMRI lies in its higher spatial resolution and wide availability to both clinical and academic researchers. Advances in technology has allowed for improvement of signal-to-noise ratio which characterizes fMRI data. This can be used for pattern classification and other statistical methods to draw increasingly complex inferences about cognitive brain states. Similar to sMRI, fMRI analyses employ the use of signal differences between states of the brain, which can be analyzed with various statistical tools, ML techniques then utilize these data to perform identification of *SZ* by comparing baseline differences. Similar to the studies using sMRI data, SVM classification has gained popularity in the past decade and has been extensively used. In the earlier days, discriminant analysis was the preferred choice of detection.

Notable work that uses fMRI data includes Calhoun et al. [67] and extended by Jafri and Calhoun [68]. In their initial work, they demonstrated on a dataset derived from 15 HC and 15 *SZ* patients, that when tasked to carry out an auditory oddball task and a Sternberg working memory task, the fMRI scan images reveal that *SZ* patients appear to “activate” less, across a smaller unique set of brain regions. This is supported by findings of reduced connectivity between joint networks made of by regions commonly classified from prevalent models of *SZ*, and henceforth initiating the use of fMRI data in many clinical studies related to *SZ*. This motivated one of the first work using fMRI data on a neural network by employing independent component analysis [68]. They managed to achieve an average accuracy of 75.6% classification by rotating the test training sets. This was significantly improved in a later study [69] using a multivariate analysis approach which successfully classified *SZ* and non-*SZ* patients with sensitivity 92% and specificity 95%. This pioneering work led to many other research work in investigating the use of other AI and ML techniques and fMRI data in classifying *SZ*, the majority of which can reach an accuracy prediction levels of Calhoun et al.

An overview of the work, sample size and accuracy from utilizing machine learning techniques on functional magnetic resonance imaging data is compiled in Table 2.

#### 3.1.3. Diffusion Tensor Imaging and Perfusion MRI

There is increasing evidence suggesting that disturbance in connectivity between different brain regions, rather than abnormalities within the brain regions themselves, are responsible for clinical symptoms and cognitive dysfunctions observed in *SZ* [107]. Thus, this led to a growing interest in WM fiber tracts, sub-serving anatomical connections between distant, as well as proximal, brain regions.

Diffusion-weighted MRI (dMRI) methods which include Diffusion Tensor Imaging (DTI) is used to map and characterize the diffusion of water as a function of spatial location in the brain. The diffusion tensor describes various measures, including magnitude, degree of anisotropy and orientation of diffusion anisotropy. The diffusion anisotropy and principal diffusion directions allows for estimates of WM connectivity patters in the brain from WM tractography. The highly sensitive changes at the cellular and microstructural level is the main contributor for the rapidly adoption of DTI, which is highly applicable in such cases. The interest in investigating disturbance in connectivity between brain regions coincides with the applicability of DTI, which makes it possible to evaluate characteristics WM fiber tracts, facilitating the process of identifying *SZ* patients [107,108].

Perfusion MRI (pMRI), on the other hand, is a non-invasive technique of obtaining measured cerebral perfusion through assessment of various hemodynamic measurements such as cerebral blood volume, cerebral blood flow, and mean transit time [109,110]. These techniques have become important clinical tools in the diagnosis and treatment of patients with cerebrovascular disease and other brain disorders, including *SZ*. Since pMRI tracks blood flow, it is also commonly used to quantify the effectiveness of drug-related pharmacological treatment for *SZ*. A summary of various studies on ML techniques on DTI and pMRI data is compiled in Table 3.

**Figure 3:**
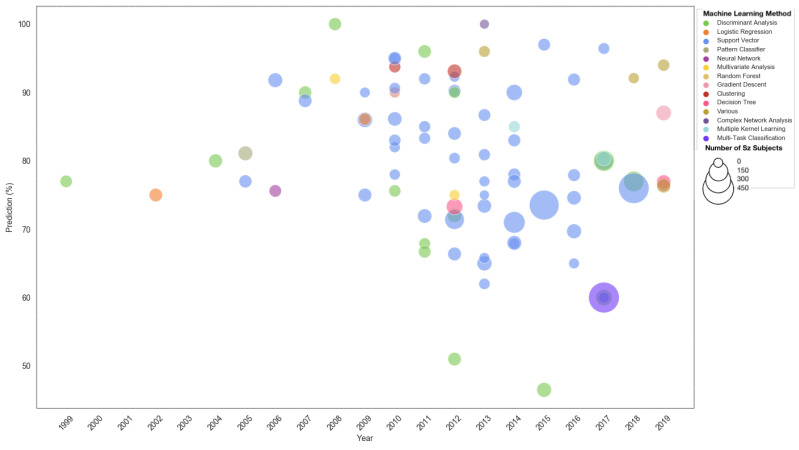
Classification by year, *SZ* sample size and prediction accuracy for the various machine learning technique for different MRI data.

Finally, we conclude this section by presenting a comparison between the different ML techniques applied to MRI data, the size of the study and the accuracy of prediction across the years in Figure 3. If more than one experiment is conducted or more than one accuracy is reported, the sensitivity prediction with the lowest accuracy will be taken for the cross-validated group.

### 3.2. Classification and Detection of SZ through Other Neurological Scans

#### 3.2.1. PET Scans

PET scans involve intrusive introduction of radioactive tracers into the subject’s bloodstream. Organs, specifically of interest in *SZ*, brain tissue, absorb the tracer, which is concentrated in areas of higher chemical activity, appearing as bright spots on the PET scan. Neuroinflammation, which is well depicted by these scans, are areas of interest as there is presence of epidemiological, genetic and clinical evidence of its involvement in *SZ*. Microglia are the resident immune cells of the central nervous system and act as major mediators of neuroinflammation. When microglia are activated, they express high levels of the 18-kDa translocator protein which can be measured in vivo with PET radio-tracers. Images collected can be used to train a ML classifier, and patterns recognized from the algorithm can then be used to predict and detect *SZ* in new subjects.

Levy et al. [117] obtained PET scan images from 12 medicated *SZ* patients and 11 HC under resting conditions and while performing a visual task. A cortical/subcortical spatial pattern was found to be significant in two directions; anterior/posterior and chiasmatic (left-anterior/right-posterior). A total of 14 two-group linear discriminant analyses were performed to classify the sample. The best individual clinical classification (Jackknife classification) occurred under visual task at two axial brain levels: at the basal ganglia (with correct classification rates of 91% specificity and 84% sensitivity), and at the cerebellum (which had rates of 82% specificity and 92% sensitivity). These high classification rates were obtained using only four coefficients of the lowest spatial frequency. These results point to the generalized brain dysfunction of regional glucose metabolism in chronic medicated schizophrenics both at rest and at a visual image-tracking task. Josin and Liddle [118] reported an analysis using a neural network to discriminate between the patterns of functional connectivity in 16 *SZ* patients and six HC. After training on data from two healthy subjects and seven *SZ* patients, the neural network successfully assigned all members of a test set of four healthy subjects and nine *SZ* patients to the correct diagnostic category. Lastly, Bose et al. [119] also tested an artificial neural network model in the discrimination of 19 *SZ* patients from 31 HC using o-dihydroxyphenylalanine (DOPA) rate constants within the anterior–posterior subdivisions of the striatum. They obtained correct classification rates of 89% sensitivity and 94% specificity. Although PET scans are reporting relatively high classification predictions of remarkable accuracy, it does not evoke confidence as means of detecting *SZ* as that current work use small sample sizes.

#### 3.2.2. EEG Signal

An electroencephalogram (EEG) is a test used to evaluate electrical activity in the brain and be used to detect certain brain disorders such as epilepsy. Event-related potentials (ERP) are obtained and analyzed. The advantage of using EEG scans stems from the ease of analysis due to its simple data type. However, EEG is not widely used for the diagnosis of mental disorders. This may be due to its low spatial resolution or depth sensitivity. Currently, there are differing views on the use of EEG as an effective tool to diagnose *SZ* [120,121,122,123,124]. In particular, it is criticized as it heavily depends on assumptions, conditions and prior knowledge regarding the patient. These may be improved through the use of data analysis and ML techniques [125]. An overview of the various study on machine learning techniques on EEG scan data is compiled in Table 4.

### 3.3. Classification and Detection of SZ through Other Techniques

The ways that genetic and DNA changes are related to *SZ* are not well understood, and the genetics of this disorder is an active area of research [135]. However, the benefit of using gene and protein data to classify *SZ* is the vast availability of data, which may propel the advancement of using ML techniques in this scope of research. There are also studies that aim to identify, classify and detect *SZ* through task-specific characteristics or non-neurological features through ML techniques. For example, cognitive and neuropsychological tests are used to examine whether neurological signs predict cognitive performance in *SZ* patients and to determine the ability of neurological signs and neuropsychological tests to discriminate *SZ* patients from healthy subjects [136,137,138,139,140]. Facial features is also an area of interest to detect *SZ* such as eye tracking [141] and facial features [142,143] as well as communication ability by tracking handwriting [144] and speech [145]. There are also traditional studies on brain shape and volume symmetry [146], signs of negative symptoms [147,148] and behavioural anomalies [149,150] as well as novel means of detecting by tracking keywords used on social media [151,152,153] or upbringing [154].

### 3.4. Composite Data Types for Classification and Detection

Since the advent of ML techniques in medical healthcare, there have been various opinions on the accuracy or the usefulness of these techniques or the type of data that gives the best prediction. These opinions are varied especially for mental health disorders [155,156,157] where the confidence interval of diagnosis by medical professionals is in itself wide. As such, some researchers have performed broad-based studies, in particular, there have been several studies that seek to compare the accuracy of specific ML technique for various types of data.

While the majority of research presented in the previous subsections generally focus on the use of just one type of data or ML technique, the question remains as to which type of data or ML technique would provide the best prediction. Hu et al. [158] was one of the few groups to implement ML algorithm as a means of performing classification by more than one type of MRI data. In particular, they employed SVM classification. Multimodal T1 structural MRI, DTI and resting-state fMRI (rs-fMRI) datasets of 10 *SZ* subjects and 10 HC were obtained. rs-fMRI and DTI datasets of subjects with mild cognitive impairment and *SZ* were then used to demonstrate their corresponding fine-granularity functional interaction (FGFI) signatures. This is done so that an examination of how FGFI features can improve the performance in the differentiation of the subject population from HC can be quantified. Consequently, with the reduced feature set, the SVM classifier was implemented to evaluate the discriminability of the FGFI features. It is seen that FGFI features yield a relatively high sensitivity 75.0% and specificity 80.0%. The ROI of this research are the left frontal, left parietal, left temporal, left occipital, right frontal, right parietal, right temporal and right occipital lobes.

Another significant work of similar nature is the research performed by Pettersson-Yeo et al. [159], however, Pettersson-Yeo et al. added non-neuroimaging data to the analysis which significantly broadened the research scope. They performed a unified study using the ML technique of SVM on genetic, sMRI, DTI, fMRI and cognitive data. Three age and gender-matched SVM paired comparison groups were created comprising 19, 19 and 15 subject pairs for first-episode psychosis (FEP) versus HC, ultra-high risk (UHR) versus HC and FEP versus UHR, respectively. Successful classification (p<0.05) comprised of the following:

- FEP versus HC: genotype, 67.86%; DTI, 65.79%; fMRI, 65.79% and 68.42%; cognitive data, 73.69%,
- UHR versus HC: sMRI, 68.42%; DTI, 65.79%, and
- FEP versus UHR: sMRI, 76.67%; fMRI, 73.33%; cognitive data, 66.67%.

The results suggest that FEP subjects are identifiable at the individual level through the use of a series of biological and cognitive measures. Comparatively, only sMRI and DTI allowed discrimination of UHR from HC subjects, thus suggesting that changes in baseline structure of WM is significant. For the first time FEP and UHR subjects have been shown to be directly differentiable at the single-subject level using cognitive, sMRI and fMRI data. The work by Pettersson-Yeo covers a series of different data types and the results support clinical development of SVM to help inform identification of FEP and UHR subjects. While this is a significant advancement in the use of ML techniques to classify patients from HC, future work is needed to provide enhanced levels of accuracy.

The works by Hu et al. and Pettersson-Yeo et al. show that there is still a huge potential for the use of AI and ML, especially with many types of data available. Just as how medical professionals use different data means to identify *SZ*, a well-trained ML model can take into account all these variables and clinical considerations to make predictions.

## 4. Outlook

As an emerging field, there remain significant gaps that can be narrowed in future research. As mentioned, the majority of papers reviewed focus on detection, with greater emphasis on using MRI data. There is significant scope to explore whether ML can have similar accuracy in the detection of *SZ* through the use of other medical data. Currently, there are few public datasets available for independent researchers to apply novel AI and ML techniques for better machine classification and detection. This important partnership between mental health and data science sectors can be beneficial to the advancement of *SZ* diagnosis. A collaborative effort to have data available could expedite research in using big data to enhance medical professionals’ experience in proper detection and diagnosis of *SZ* in potential patients.

Furthermore, while there is a fair number of studies that focused on treatment and support for patients with *SZ*, comparatively fewer research has explored applications in support domains such as education, public health, research and clinical administration. This forms a large area for innovating, particularly when leveraged by ML techniques as it contributes a significantly large volume of data that can be utilized in further coordination such as public mental health education, big data research and clinical administration. One possible concern is the emergence of cyber risks when integrating AI, ML, and big data into healthcare infrastructure. However, with the development of technology, also comes an active and advancing field of research [160,161,162] that seeks to mitigate cyber risks to protect healthcare givers and patients from the small risks that come with the wide opportunities made available with technological integration. With proper intervention, these risks could be mitigated.

Current research and the choice of supervised learning ML techniques (SVM, k-nearest neighbours, decision trees, regression etc.) is indicative of the focus on detection. Supervised learning is typically designed using large, retrospective, labelled datasets ideal for classification tasks. Future researchers could consider the possibility of using less structured, prospective data for real-time ML analysis. While such studies cannot replace the emotive aspect of physician-patient connection, advances in these analytic unsupervised or online learning may enable researchers and clinicians to provide personalized and context-sensitive information for assessment. This can also alleviate the main issues, such as the quality of data, that hinder the effectiveness of many supervised learning ML models.

We caution that ML should not replace other research or analytic approaches; rather, it complements and value-add to *SZ* research. While the question of which ML technique or data type is most reliable or most accurate depends heavily on the study and nature of the data collected, it does show that different research groups can produce a detection mechanism of an acceptable classification accuracy. The push for a data-driven research through means of using ML techniques may require greater collaboration between research institutions and healthcare bodies to harmonize and share data, in a responsible and sensitive manner. These forms of collaboration seek to maximize the effectiveness and accuracy of the models developed. Thus, the emerging question should not be about which data type is best or which ML technique is the best. These are questions of the past as we have seen that regardless of data type, various ML techniques have proven to have high prediction accuracy. Furthermore, the data inputs are from different sources and quality. A step towards the future should be to build a learning model that can receive comprehensive types of data to make better predictions through a combination of multiple ML techniques rather than solely relying on a single data type or ML technique. This, coupled with a centralized standard of data curation for clinical and academic researchers would create a level platform for providing a basis for comparison of data type and technique. Researchers and medical professionals who wish to implement and integrate AI and ML techniques, may refer to the survey conducted by Coronato et al. [163,164].

Finally, while still debated, the successful and competitive prediction accuracy motivate the employment of ML techniques to evaluate effectiveness of pharmacological treatment. To date, *SZ* remains a complex disorder which requires prompt therapy upon detection of early signs of psychotic episodes. Medical professionals must consider many factors while developing a comprehensive and effective treatment plan. These considerations can be aided by the advent of ML techniques in optimizing treatment through pharmacological options. This is one of the motivations to use AI and ML algorithms for the purpose of detection and quantifying treatment aid in the eventual goal of enhancing translational medicine for individualized management of *SZ* patients. This, however, cannot overwrite on-going research in non-pharmacological treatment, which fundamentally remains an important pillar to mental health treatment.

## 5. Conclusions

This review is in line with the growing interest of applying ML to areas of mental health research. The current work focus on detecting and classifying *SZ* by quantifying them according to the AI techniques and machine learning algorithms. We formally synthesized and consolidated the literature on ML and big data with application to *SZ* by highlighting the advances in current research and applications in practice. The dominant work in current research has focused on the benefits of ML as a means to improve detection and diagnosis of *SZ*. The studies presented in this review demonstrate the need to push the boundaries of AI and ML in the healthcare profession, indicating the potential of using computers as a means of enhancing capabilities in dealing with *SZ* diagnosis.

Research in the field of AI and ML for *SZ* has revealed exciting advances. The work reviewed shows that ML can contribute in the area of detection and diagnosis of *SZ* conditions. Research into treatment and support has demonstrated initial positive results. The need for more comparative studies that uses composite data and analyzed with multiple ML techniques, we highlight the work presented by Hu et al. and Pettersson-Yeo et al. In their work, they concluded that FEP subjects are identifiable through the use of biological and cognitive measures, while sMRI and DTI is particularly useful in differentiating high-risk patients with healthy subjects. They were able to come to this conclusion because of their extensive use of data types and AI techniques. With ML tools becoming more accessible for researchers and clinicians, it is expected that the field will continue to grow and that novel applications for detection and pharmacological treatment with the help of advanced AI and ML techniques will follow. More information please see Supplementary Materials.

## Figures and Tables

**Table 1:** Summary of work and predictions relating to the detection of *SZ* using data from structural MRI scans via various artificial intelligence techniques and machine learning algorithms.

Study	Year	Subjects		Prediction	AI/ML Technique
		Patients	Control		
Leonard et al. [26]	1999	37♂	33♂	77%	Linear Discriminant Function Analysis (DFA)
Csernansky et al. [31]	2002	52	65	75% (sensitivity) 76.9% (specificity)	Logistic Regression Model
Nakamura et al. [33]	2004	30♂, 27♀	25♂, 22♀	80%♂, 81.6%♀	DFA
Yushkevich et al. [34]	2005	46	46	72% (sensitivity) 70% (specificity)	Support Vector Machine (SVM)
Davatzikos et al. [32]	2005	69	79 (matched)	81.1% (mixed) 85%♂, 82%♀	High-dimensional nonlinear Pattern Classifier
Fan et al. [35]	2006	23♀, 46♂	38♀, 41♂	91.8%♀, 90.8%♂	Nonlinear SVM, leave-one-out cross-validation
Yoon et al. [36]	2007	21♀, 32♂	52 (matched)	at least 88.8%	SVM, PCA
Kawasaki et al. [37]	2007	30♂, 16♂	30♂, 16♂	90%, 80%, 75% (Jackknife)	Multivariate Linear DFA, Jackknife approach
Castellani et al. [38]	2009	54	54	up to 75% and 85% (sex stratified)	Scale Invariance Feature Transform (SIFT), SVM
Pohl and Sabuncu [39]	2009	16	17 (age-matched)	up to 90%	Linear SVM, Leave-one-out cross-validataion
Sun et al. [40]	2009	36	36 (sex- and age-matched)	86.1%	Pattern Classification Analysis with Sparese Multi-nomial Logistic Regression Classifier, Leave-on-out cross-validation
Koutsouleris et al. [27]	2009	A1: 20 (ARMS-E), 25 (ARMS-L) A2: 15 (ARMS-T), 18 (ARMS-NT)	A1: 25 (matched) A2: 17 (matched) Cross-validation: 45	at least 86% (sensitivity) at least 93% (specificity)	SVM, Multivariate Pattern Analysis (MVPA)
Takayanagi et al. [41]	2010	17♂, 17♀	24♂, 24♀	75.6%, 82.9%	Linear DFA
Castellani et al. [42]	2010	64	60	up to 86.13%	SVM
Koutsouleris et al. [43]	2010	25	28	83%	SVM with Partial-least-squares Pattern Analysis
Kasparek et al. [44]	2011	39	39	66.7% (sensitivity) 76.9% (specificity)	Maximum-uncertainty Linear Discriminant Analysis (MLDA)
Karageorgiou et al. [45]	2011	28	47	67.9% (sensitivity) 72.3% (specificity) using PCA-LDA (sMRI only)	LDA, Principal Component Analysis (PCA)
Castellani et al. [46]	2011	30	30	up to 83.33%	SVM, Leave-one-out cross-validation
Ulaş et al. [47]	2011	64	60	71.93% (SVM)	1-Nearest Neighbour, Linear SVM
Koutsouleris et al. [48]	2012	16/21	22	92.3% 66.9% 84.2%	SVM
Castellani et al. [49]	2012	54	54 (matched)	at least 66.38%	SIFT and nonlinear SVM
Nieuwenhuis et al. [50]	2012	128, 155	111, 122	71.4%, 70.4%	SVM, Leave-one-out cross-validation
Ulaş et al. [28]	2012	50	50	84% (MKL) 77% (SVM)	SVM, MKL
Ulaş et al. [29]	2012	21♂, 21♀	19♂, 21♀	90.24% (CLMKL) 71.95% (SVM)	SVM, Clustered Localized MKL (CLMKL)
Ota et al. [51]	2012	38♀, 23♀	105♀, 23♀	74% (sensitivity) 70% (specificity)	DFA
Bansal et al. [52]	2012	65	40	93.1% (sensitivity) 94.5% (specificity)	Hierarchical clustering, Split-half and Leave-one-out cross-validation
Greenstein et al. [53]	2012	98	99	73.3%	Random Forest
Borgwardt et al. [54]	2013	16/23	22	86.7% 80.7% 80.0%	SVM, Nested cross-validation
Iwabuchi et al. [55]	2013	19	20	up to 77%	SVM
Zanetti et al. [56]	2013	62	62 (matched)	73.4%	SVM
Gould et al. [57]	2014	126/74	134	71%	SVM
Perina et al. [58]	2014	21♂, 21♀	19♂, 21♀	83% (sensitivity)	SVM
Schnack et al. [59]	2014	46/47	43	90%	SVM
Cabral et al. [60]	2016	71	74	69.7%	SVM, MVPA
Lu et al. [61]	2016	41	42 (sex- and age-matched)	91.9% (sensitivity) 84.4% (specificity)	SVM, Recursive Feature Elimination (RFE)
Yang et al. [30]	2016	40	46	77.91%	MLDA, SVM
Squarcina et al. [62]	2017	127	127	80%	SVM
Rozycki et al. [63]	2018	440	501	76%	Linear SVM
de Moura et al. [64]	2018	143, 32	82	77.6% (sensitivity) 68.3% (specificity)	MLDA
Liang et al. [65]	2019	98, 54	106, 48	75.05%, 76.54%	Gradient Boosting Decision Tree
Deng et al. [66]	2019	65	60	76.9% (sensitivity) 75.0% (specificity)	Random Forest

**Table 2:** Summary of work and predictions relating to the detection of *SZ* using data from functional MRI scans via various artificial intelligence techniques and machine learning algorithms.

Study	Year	Subjects		Prediction	AI/ML Technique
		Patients	Control		
Jafri and Calhoun [68]	2006	38	31	75.6%	Neural network
Calhoun et al. [69]	2008	21	26	92% (sensitivity) 95% (specificity)	MVPA
Anderson et al. [70]	2010	14	6	up to 90%	Multivariate Random Forest
Arribas et al. [71]	2010	21	25	90%	Stochastic Gradient Learning based on minimization of Kullback-Leibler divergence
Shen et al. [72]	2010	32	20	93.75% (sensitivity) 75% (specificity)	Low-dimensional embedding and self-organized C-means clustering
Yang et al. [73]	2010	20	20	at least 82% (using fMRI data)	SVM
Castro et al. [74]	2010	52	54	95%	Composite kernels, Linear and Gaussian SVM, Leave-two-out cross-validation
Costafreda et al. [75]	2011	32	40	92% (seonsitivity)	SVM
Fan et al. [76]	2011	31	31	up to 85.5%	SVM, Linear kernel, Radial basis function kernel, Sigmoid kernel
Du et al. [77]	2012	28	28	90%	Fisher’s linear discriminant analysis, Default mode network, Majority vote, Leave-one-out cross-validation
Liu et al. [78]	2012	25	25 (siblings) 25 (HC)	80.4% (*SZ* vs. HC)	Nonlinear SVM with polynomial kernel
Venkataraman et al. [79]	2012	18	18	75%	Multivariate classification
Yoon et al. [80]	2012	51	51 (age-matched)	51.0% (sensitivity) 64.7% (specificity)	Linear DFA, Leave-one-out cross-validation
Anderson and Cohen [81]	2013	74	72	65%	SVM
Arbabshirani et al. [82]	2013	28	28	up to 96% (KNN)	Various (10 types) linear and nonlinear classifier
Fekete et al. [83]	2013	8♂	10♂	100%	Complex network analysis, Block diagonal optimization.
Yu et al. [84]	2013	24	25 (siblings) 22 (matched HC)	62%	SVM, PCA, Leave-one-out cross-validation
Yu et al. [85]	2013	32 (*SZ*) 19 (Depression)	38	80.9%	SVM, Intrinsic DA, Leave-one-out cross-validation
Anticevic et al. [86]	2014	Sample: 90 Validation: 23	Sample: 90 (matched) Validation: 23 (matched)	Sample: 75.5% (sensitivity), 72.2% (specificity) Validation: 67.9% (sensitivity), 77.8% (specificity)	Linear SVM, Leave-one-out cross-validation
Brodersen et al. [87]	2014	41	42	78%, 71%	Linear SVM, Variational Bayesian Gaussian mixture
Castro et al. [88]	2014	31	21	90% (L-norm MKL), 85% (Lp-norm MKL)	L-norm and Lp-norm MKL
Guo et al. [89]	2014	69	62	68%	SVM
Watanabe et al. [90]	2014	54	67	at least 77.0%	Fused Lasso and GraphNet regularized SVM
Cheng et al. [91]	2015	415	405	73.53–80.92%	SVM
Chyzhyk et al. [92]	2015	26/14	28	97–100%	Linear SVM
Kaufmann et al. [93]	2015	71	196	46.5% (sensitivity) 86.0% (specificity)	Regularized LDA, Leave-one-out cross-validation
Pouyan and Shahamat [94]	2015	10	10	up to 100% (sensitivity and specificity)	ICA, PCA, Various, Leave-one-out cross-validation
Mikolas et al. [95]	2016	63	63 (sex- and age-matched)	74.6% (sensitivity) 71.4% (specificity)	Linear SVM
Peters et al. [96]	2016	18	18	up to 91%	SVM, Leave-one-out cross-validation
Yang et al. [30]	2016	40	40	77.91%	MLDA, SVM
Skaatun et al. [97]	2017	182	348	up to 80%	Multivariate regularized LDA
Chen et al. [98]	2017	20 (*SZ*) 20 (depression)	20	60% (sensitivity) 90% (specificity)	Linear SVM, MVPA
Kaufmann et al. [99]	2017	90 (*SZ*) 97 (bipolar)	137 (HC)	60% (sensitivity) 90% (specificity)	5-class regularized LDA, k-fold cross-validation model
Guo et al. [100]	2017	28	28 family-based control (FBC) 40 (HC)	SVM: 96.43% (sensitivity) 89.29% (specificity, FBC)	SVM, Receiver operating characteristic (ROC) curve
Iwabuchi and Palaniyappan [101]	2017	71	62	80.32%	MKL
Yang et al. [102]	2017	446	451	60–86%	Multi-task classification, 10-fold cross-validation
Bae et al. [103]	2018	21	54	92.1% (SVM)	Various (5 types), 10-fold cross-validation
Li et al. [104]	2019	60	71	76.34% (LDA)	KNN, Liner SVM, Radial basis SVM, LDA
Chatterjee et al. [105]	2019	34	34	94% (SVM) 96% (1-NN)	SVM, k-nearest neighbours
Kalmady et al. [106]	2019	81	93 (sex- and age-matched)	87%	L2-regularized Logistic regression

**Table 3:** Summary of work and predictions relating to the detection of *SZ* using data from diffusion-weight MRI, diffusion tensor imaging and perfusion MRI scans via various artificial intelligence techniques and machine learning algorithms.

Study	Year	Subjects		Prediction	AI/ML Technique
		Patients	Control		
Caan et al. [111]	2006	34♂	24	(not reported)	LDA, PCA
Caprihan et al. [112]	2008	45	45 (age-matched)	100%	DPCA
Ingalhalikar et al. [113]	2010	27♀	37♀	90.62%	Nonlinear SVM
Rathi et al. [114]	2010	21 (FEP)	20 (age-matched)	SH: 78% (sensitivity) 80% (specificity) F2T: 86% (sensitivity) 85% (specificity)	K-nearest neighbours, Parzen window classifier, SVM
Ardekani et al. [115]	2011	50	50 (age- and sex-matched)	FA: 96% (sensitivity) 92% (specificity) MD: 96% (sensitivity) 100% (specificity)	Fisher’s LDA
Squarcina et al. [116]	2015	35 (FEP)	35	83%	SVM

**Table 4:** Summary of work and predictions relating to the detection of *SZ* using data from electroencephalogram scans via various artificial intelligence techniques and machine learning algorithms.

Study	Year	Subjects		Prediction	AI/ML Technique
		Patients	Control		
Knott et al. [126]	1999	14	14	at least 89.3%	DFA, Jackknife classification
Neuhaus et al. [127]	2011	40	40 (matched)	79.9% (balanced)	SVM (linear, quadratic and radial basis kernels), LDA, Quadratic discriminant analysis (QDA), KNN, naïve Bayes with equal and unequal variances and Mahalanobis classification
Iyer et al. [128]	2012	13	20	max 76% (ensemble averaging) 100% (single-trial)	Random Forest, 10-fold stratified cross-validation
Laton et al. [129]	2014	54	54 (sex- and age-matched)	up to 84.7%	Naïve Bayes, SVM and decision tree, with two of its improvements: adaboost and Random Forest
Neuhaus et al. [130]	2014	144	144 (matched)	74% (balanced)	LDA and QDA (with their diagonal variants), SVM (linear, polynomial, radial basis and multilayer perceptron kernels), Naïve Bayes, KNN (Euclidean and cosine distance measures) and Mahalanobis classification
Johannesen et al. [131]	2016	40	12	up to 87%	1-norm SVM
Shim et al. [132]	2016	34	34	Maximum: 88.24% (combined) 80.88% (sensor-level) 85.29% (source-level)	SVM, Leave-one-out cross-validation
Taylor et al. [133]	2017	21	22	80.84%	SVM, Gaussian processes classifiers, MVPA
Krishnan et al. [134]	2020	14	14 (sex- and age-matched)	up to 93%	Various, SVM (Radial Basis Function)

## Data Availability

Not applicable.

## Supplementary Materials

The following are available online at https://www.mdpi.com/article/10.3390/ijerph18116099/s1, Table S1: Summary of work relating to the detection of *SZ* using data from structural MRI scans via various artificial intelligence techniques and machine learning algorithms. Table S2: Summary of work relating to the detection of *SZ* using data from functional MRI scans via various artificial intelligence techniques and machine learning algorithms. Table S3: Summary of work relating to the detection of *SZ* using data from diffusion-weight MRI, diffusion tensor imaging and perfusion MRI scans via various artificial intelligence techniques and machine learning algorithms. Table S4: Summary of work relating to the detection of *SZ* using data from electroencephalogram scans via various artificial intelligence techniques and machine learning algorithms.
