# Supplementary material for: Schizophrenia: A Survey of Artificial Intelligence Techniques Applied to Detection and Classification
Source: Int J Environ Res Public Health. 2021 Jun 5;18(11):6099. doi: 10.3390/ijerph18116099 (PMC8201065; doi:10.3390/ijerph18116099)
Supplement: Supplementary file 1 [file ijerph-18-06099-s001.zip › ijerph-1226438-supplementary.pdf]

Supplementary Information: Summary Table

The following information is obtained directly from the reviewed papers to give readers an overview of the methodology adopted by the various studies. This should be read together with the data presented in the corresponding tables in the main text.

Table 1

Study	Year	Methodology	AI/ML
Leonard *et al.*	1999	This is the first study to demonstrate that schizophrenics can be distinguished from matched controls on the basis of brain anatomy alone.	Linear Discriminant Function Analysis (DFA)
Csernansky *et al.*	2002	T1-weighted MRI were collected in 52 schizophrenia and 65 comparison subjects who were similar in age, gender, and parental socioeconomic status. Log-likelihood ratio values were derived from the linear combination of eigenvectors 1, 5, 7, 14, and 15, which yielded the maximal between-group discrimination in a logistic regression model. Significant abnormalities of hippocampal shape and asymmetry (but not volume after total cerebral volume was included as a covariate) were found in the *SZ* subjects. However, the magnitude of these deformities were not related to severity or duration of illness.	Logistic Regression Model
Nakamura *et al.*	2004	Significant enlargements of the left inferior horn, left and right sylvian fissure, along with significant volume reduction of right temporal lobe were observed in female patients.	DFA
Yushkevich *et al.*	2005	ROI: third ventricle, right thalamus	Support Vector Machine (SVM)
Davatzikos *et al.*	2005	Neuroleptic-naïve and previously treated patients with DSM-IV characterised *SZ* and sociodemographically similar HC were studied. sMRI showed reduced GM and increased ventricular cerebrospinal fluid volumes in patients with *SZ* in the whole brain and in specific foci: hippocampus and adjacent WM, cingulate and orbitofrontal cortex, frontotemporal and parietotemporal areas, and occipital areas near the lingual gyrus.	High-dimensional nonlinear Pattern Classifier
Fan *et al.*	2006	Regions that display strong correlations between tissue volume and classification (clinical) variables are extracted using a watershed segmentation algorithm, taking into account the regional smoothness of the correlation map which is estimated by a cross-validation strategy to achieve robustness to outliers. A volume increment algorithm is then applied to these regions to extract regional volumetric features, from which a feature selection technique using nonlinear SVM-based criteria is used to select the most discriminative features, according to their effect on the upper bound of the leave-one-out generalization error. Finally, SVM-based classification is applied using the best set of features, and it is tested using a leave-one-out cross-validation strategy.	Nonlinear SVM, leave-one-out cross-validation
Yoon *et al.*	2007	52 healthy subjects were matched with the patient group for age, sex, handedness, and socioeconomic status. Discriminative patterns derived at every vertex in the original feature space with respect to SVM were analysed with definitive findings of brain abnormalities in schizophrenia for establishing practical confidence. This study showed that some principal components might be more useful than others for classification, but not necessarily matching the ordering of the variance amounts they explained.	SVM, PCA
Kawasaki *et al.*	2007	Analysis performed on two, all-male, cohorts with varying classification rates, 90% and 80%, respectively. Another approach, the Jackknife approach was also used on the first cohort.	Multivariate Linear DFA, Jackknife approach
Castellani *et al.*	2009	Scale Invariance Feature Transform (SIFT) was used with SVM, characterised by the local region descriptors. Then, matching is obtained by introducing the local kernel for which the samples are represented by unordered set of features. A new weighting approach is proposed to take into account the discriminative relevance of the detected groups of features. Preliminary results on Dorsolateral Pre-Frontal Cortex region reports up to 75% of successful classification rate with this technique and the performance has improved up to 85% when the subjects have been stratified by sex (females).	Scale Invariance Feature Transform (SIFT), SVM
Pohl and Sabuncu	2009	Method estimates the structure-specific transformation parameters directly from medical scans by minimizing a Kullback-Leibler divergence measure. The resulting parameters are then input to a linear SVM classifier, which assigns individual scans to a specific clinical group. ROI in both hemispheres: superior temporal gyrus, amygdala, hippocampus, and para-hippocampal gyrus. On this small size dataset, the approach performs classification based on the MRI directly.	Linear SVM, Leave-one-out cross-validataion
Sun *et al.*	2009	Three-dimensional cortical GM density maps were generated for 36 patients with recent-onset psychosis and 36 sex- and age-matched control subjects using a cortical pattern matching method. Next, the sparse multi-nomial logistic regression classifier was applied to the cortical GM density maps to discriminate psychotic patients from HC. Patients showed significantly lower GM density, particularly in prefrontal, cingulate, and lateral temporal brain regions.	Pattern Classification Analysis with Sparse Multi-nomial Logistic Regression Classifier, Leave-on-out cross-validation

Continued on next page

Table 1

Study	Year	Methodology	AI/ML
Koutsouleris *et al.*	2009	Multivariate neuroanatomical pattern classification was performed on the sMRI data of individuals in ARMS (early and late) vs HC. The predictive power of the method was then evaluated by categorizing the baseline imaging data of individuals with transition to psychosis vs those without transition vs HC after 4 years of clinical follow-up. Classification generalizability was estimated by cross-validation and by categorizing an independent cohort of 45 new HC. For the first analysis (HC vs ARMS-E vs ARMS-L), a sample of 25 HC (HC1) was randomly selected from a previously described group of 75 HC to create a balanced design regarding group sizes. The HC1 group was matched groupwise for age, handedness, and years of education to the ARMS-E and ARMS-L samples. The first classification analysis included 20 early and 25 late at-risk individuals (28 men and 17 women) and 25 matched HC. The second analysis consisted of 15 individuals with transition, 18 without transition, and 17 matched HC. A second matched HC sample was used for the second analysis (HC vs ARMS-T vs ARMS-NT) by randomly removing 8 subjects from HC1. Finally, 45 new HC were recruited for the external validation of these classification analyses. The 3-group, cross-validated classification accuracies of the first analysis were 86% (HC vs the rest), 91% (early at-risk individuals vs the rest), and 86% (late at-risk individuals vs the rest). The accuracies in the second analysis were 90% (HC vs the rest), 88% (individuals with transition vs the rest), and 86% (individuals without transition vs the rest). Independent HC were correctly classified in 96% (first analysis) and 93% (second analysis) of cases.	SVM, Multivariate Pattern Analysis (MVPA)
Takayanagi *et al.*	2010	Twenty nine ROI were measured on 1-mm-thick coronal slices from the prefrontal and central parts of the brain.	Linear DFA
Castellani *et al.*	2010	The approach is inspired by natural language processing. Local brain surface geometric patterns are quantised to visual words, and their co-occurrences are encoded as visual topic. To do this, a generative model, the probabilistic Latent Semantic Analysis is learned from quantised shape descriptors (visual words). Finally, a generated score is extracted from the learned models, used as input of a SVM. ROI: left-amygdala.	SVM
Koutsouleris *et al.*	2010	Deformation-based morphometry and partial-least-squares revealed a pattern of pronounced morphometric changes in ARMS versus HC that affected predominantly the right prefrontal, as well as the perisylvian, parietal and periventricular structures, and that was more pronounced in the converters versus the non-converters. The SVM analysis facilitated a reliable prediction of these longitudinal brain changes in individual out-of training cases by relying on baseline patterns for that involved ventricular enlargements, as well as prefrontal, perisylvian, limbic, parietal and subcortical volume reductions.	SVM with Partial-least-squares Pattern Analysis
Kasparek *et al.*	2011	Patients were first-episode schizophrenia patients. The method used as the maximum-uncertainty linear discrimination analysis (MLDA) of MRI brain intensity images. The rate of correct classifications of patients with poor and good outcomes was analysed using chi-square tests. MLDA classification was significantly better than classification by chance.	Maximum-uncertainty Linear Discriminant Analysis (MLDA)
Karageorgiou *et al.*	2011	sMRI measurements and nucleotide polymorphism (NP) test results from patients and healthy subjects were used. The accuracy of classification was compared between using NP only, sMRI only and sMRI-NP using two methods (a) Stepwise (STP) Linear Discriminant Analysis (LDA), and (b) LDA through Principal Component Analysis (PCA). The analysis produced the following results<br>• NP only: (a) STP-LDA: 71.4% sensitivity and 80.9% specificity, (b) PCA-LDA: 78.5% sensitivity and 91.5% specificity.<br>• sMRI only: (a) STP-LDA: 64.3% sensitivity and 76.6% specificity, (b) PCA-LDA: 67.9% sensitivity and 72.3% specificity.<br>• sMRI-NP: (a) STP-LDA: 64.3% sensitivity and 83.0% specificity, (b) PCA-LDA: 89.3% sensitivity and 93.6% specificity.	LDA, Principal Component Analysis (PCA)
Castellani *et al.*	2011	A new shape descriptor was able to encode morphometric properties of a brain image or region using diffusion geometry techniques based on the local Heat Kernel. Using this approach, it is possible to design a versatile signature, employed in this case to classify normal subjects and patients affected by schizophrenia. ROI: left-thalamus.	SVM, Leave-one-out cross-validation
Ulaş *et al.*	2011	Classified patients affected by *SZ* by means of dissimilarity-based classification techniques was applied to brain MRI. Instead of working with features directly, pairwise distances between expert delineated ROIs were considered as representations based upon which learning and classification can be performed.	1-Nearest Neighbour, Linear SVM
Koutsouleris *et al.*	2012	MRI-based classification system using SVM was used in predicting disease conversion in at-risk individuals involving MRI data of 22 healthy volunteers and 16/21 at-risk subjects with/without a subsequent disease conversion. Diagnostic performance was measured in unseen test cases using repeated nested cross-validation. The classification accuracy in the HC vs converter, HC vs nonconverter, and converter vs nonconverter analyses were 92.3%, 66.9%, and 84.2%, respectively.	SVM
Castellani *et al.*	2012	The objective of this study was to use a combined local descriptor, namely SIFT and a nonlinear SVM technique to automatically classify patients with *SZ*. The dorsolateral prefrontal cortex (DLPFC) was chosen as the ROI. Substantial results were obtained for the classification of the whole dataset (left side 75%, right side 66.38%). The performances were higher when females (left side 84.09%, right side 77.27%) and seniors (left side 81.25%, right side 70.83%) were considered separately. No effects of age, gender, anti-psychotic treatment and chronicity were shown on DLPFC volumes.	SIFT and nonlinear SVM
Nieuwenhuis *et al.*	2012	Using SVM, the model was built from 128 patients and 111 HC and classified 71.4% correct (leave-one-out). The result was replicated and validated by testing on an independent sample of 155 patients and 122 HC, with classification rate of 70.4%. The model's discriminative pattern showed that GM density decreases in frontal, superior temporal lobes and hippocampus in *SZ* patients with respect to HC and increases in GM density in basal ganglia and left occipital lobe.	SVM, Leave-one-out cross-validation

Continued on next page

Table 1

Study	Year	Methodology	AI/ML
Ulaş *et al.*	2012	Technique uses multiple kernel learning (MKL) from eight ROIs. Giving more accurate prediction than using single source SVMs, feature concatenation and kernel averaging but also evaluated the relevance of the brain biomarkers in predicting this disease. On a dataset of 50 patients and 50 healthy controls an increase of 7% accuracy (84%) compared to SVM was achieved.	SVM, MKL
Ulaş *et al.*	2012	Proposed a Clustered Localised Multiple Kernel Learning (CLMKL) algorithm by encoding in the classication model the information on the clusters of apriory known stratifications. In this work, there were three ROIs from the two hemispheres of the brain (left and right) dorsolateral prefrontal cortex, entorhinal cortex, and thalamus were found to be impaired in *SZ* patients. Accuracies on schizophrenia detection dataset using all ROIs. SVM alone 71.95%, CLMKL 90.24%.	SVM, Clustered Localised MKL (CLMKL)
Ota *et al.*	2012	Patients and healthy subjects were evaluated using optimised voxel-based morphometry, then, a discriminant analysis reflecting the results of this evaluation was adopted.	DFA
Bansal *et al.*	2012	Automated method to diagnose individuals as having one of various neuropsychiatric illnesses using only sMRI scans. The method employs a semi-supervised learning algorithm that discovers natural groupings of brains based on the spatial patterns of variation in the morphology of the cerebral cortex and other brain regions. Split-half and leave-one-out cross-validation analyses in large MRI datasets was used to assess the reproducibility and diagnostic accuracy of those groupings. Hierarchical clustering procedures to discover naturalistic groupings of brain features was employed.	Hierarchical clustering, Split-half and Leave-one-out cross-validation
Greenstein *et al.*	2012	Using 74 sMRI subregions and Random Forest (RF), this work classified 98 childhood onset *SZ* (COS) patients and 99 age, sex, and ethnicity-matched HC. RF was also used to estimate the probability of being classified as a *SZ* patient based on MRI measures. Brain regions jointly classified COS and HC with 73.7% accuracy. The regions that were most important in classifying groups included left temporal lobes, bilateral dorsolateral prefrontal regions, and left medial parietal lobes.	Random Forest
Borgwardt *et al.*	2013	Classifying disease stages from 16 at-risk individuals with subsequent transition to psychosis (ARMS-T) and 23 patients with FEP. Pairwise and multigroup biomarkers were constructed using the sMRI data. The performance of these biomarkers was measured in unseen test cases using repeated nested cross-validation. The classification accuracies in the HC vs FEP, HC vs ARMS-T, and ARMS-T vs FEP analyses were 86.7%, 80.7%, and 80.0%, respectively. The neuroanatomical decision functions underlying these discriminative results particularly involved the frontotemporal, cingulate, cerebellar, and subcortical brain structures.	SVM, Nested cross-validation
Iwabuchi *et al.*	2013	Evaluated the classifier performance using back-to-back sMRI in two field strengths (3- and 7-T) to discriminate patients with schizophrenia from healthy controls. GM and WM images were used as inputs into a SVM to classify patients and control subjects. Seven Tesla classifiers outperformed the 3-T classifiers with accuracy reaching as high as 77% for the 7-T GM classifier compared to 66.6% for the 3-T GM classifier.	SVM
Zanetti *et al.*	2013	There were 62 age, gender and educationally-matched HC. A SVM classifier was employed with the following purposes: 1) to ascertain how distinguishable are *SZ* individuals from healthy controls at the time of FEP using T1-weighted MRI data; 2) to evaluate the performance of the classifier in correctly predicting 1-year outcome of FEP; and 3) to describe patterns of complex morphological features significantly associated with *SZ* at an early course of the illness. The spatial map discriminating FEP from HC revealed a complex pattern of regional volumetric abnormalities in the former group, affecting fronto-temporal-occipital grey and white matter regions bilaterally, including the inferior fronto-occipital fasciculus, as well as the third and lateral ventricles. However, the classifier failed to predict 1-year prognosis of first-episode *SZ* (58.3%).	SVM
Gould *et al.*	2014	SVM classification of grey and white matter volume data from 126 *SZ* patients previously allocated the cognitive spared subtype, 74 cognitive deficit *SZ* patients, and 134 HC. Using this method, cognitive subtypes were distinguished from HC with up to 72% accuracy. Cross-validation analyses between subtypes achieved an accuracy of 71%, suggesting that some common neuroanatomical patterns distinguish both subtypes from HC. Notably, cognitive subtypes were best distinguished from one another when the sample was stratified by sex prior to classification analysis: <60% without stratification and increased to 83% for females with sex stratification.	SVM
Perina *et al.*	2014	This paper exploits the embedding provided by the counting grid model and proposes a framework for the classification and the analysis of brain MRI images. A generative kernel based on the diffusion distance in the counting grid space is introduced, which reached 83% accuracy in the classification of *SZ* patients. Finally, in order to implement the hybrid generative-discriminative scheme the proposed generative kernel is employed with a SVM as a discriminative classifier.	SVM
Schnack *et al.*	2014	Images were obtained from 66 *SZ* patients, 66 patients with bipolar disorder and 66 HC on a 1.5 T MRI scanner. Three SVM were trained to separate patients with *SZ* from HC, patients with *SZ* from those with bipolar disorder, and patients with bipolar disorder from HC, respectively, based on their GM density images. The predictive power of the models was tested using cross-validation and in an independent validation set of 46 *SZ* patients, 47 patients with bipolar disorder and 43 HC scanned on a 3 T MRI scanner. *SZ* patients could be separated from HC with an average accuracy of 90%. Application of the 1.5 T models on the 3 T validation set yielded average classification accuracies of 76% (HC vs *SZ*), 66% (bipolar vs *SZ*) and 61% (HC vs bipolar).	SVM
Cabral *et al.*	2016	This study aimed to investigate effects of clinical and sociodemographic variables on the classification by applying multivariate pattern analysis to both GM volume and functional-connectivity measures in patients with *SZ* and HC. The dependence of neurodiagnostic performance on clinical and sociodemographic variables was evaluated. The resting-state (RS) fMRI classifier, showed a slightly higher accuracy (70.5%) compared to the sMRI classifier (69.7%). The combination of sMRI and RS outperformed single MRI modalities classification by reaching 75% accuracy. In addition the results of RS based moderator analysis showed that age of patients, as well as their age at the illness onset were the most important clinical features.	SVM, MVPA
Lu *et al.*	2016	A combined SVM with recursive feature elimination (RFE) was used. Employed voxel-based morphometry analysis to compare GM volume and WM volume between 41 *SZ* patients and 42 sex- and age-matched HC. *SZ* patients showed GM and WM abnormalities in several brain structures primarily involved in the emotion, memory and visual systems.	SVM, Recursive Feature Elimination (RFE)

Continued on next page

Table 1

Study	Year	Methodology	AI/ML
Yang *et al.*	2016	28 resting-state networks were first identified by group independent component analysis. Functional network connectivity was then calculated as the Pearsons correlation coefficient between any pair of resting-state network time courses, and 378 between-network connectivities were obtained. Additionally, 32 anatomical features were identified from anatomical data through independent component analysis. In a further analysis, the overall functional and structural features were combined in conjunction with a SVM to discriminate patients with SZ from HC. Maximum-uncertainty linear discriminant analysis was introduced to extract highly discriminative features. Using classifier with combined features of sMRI and fMRI data achieved higher accuracy than the single-modal features (accuracy 77.91% vs 72.09%).	MLDA, SVM
Squarcina *et al.*	2017	A method based on MKL was developed, exploits the effect of these confounding variables with a subject-depending kernel weighting procedure. ROI: frontal and temporal areas.	SVM
Rozycki *et al.*	2018	Using advanced multi-variate analysis tools and pooled data from casecontrol imaging studies conducted, a neuroanatomical signature of patients with SZ was found, and its robustness and reproducibility across sites, populations, and scanners, was established for single-patient classification. Multivariate classification using pooled data via linear SVM achieved a cross-validated prediction accuracy of 76%.	Linear SVM
de Moura *et al.*	2018	Psychiatrists assessed 82 HC, 143 patients with chronic SZ from an outpatient unit and 32 FEP from the psychiatric emergency unit. FEP group has a maximum-uncertainty linear discriminant analysis score distribution similar to patents with SZ than the healthy control group.	MLDA
Liang *et al.*	2019	This study used Gradient Boosting Decision Tree to identify the most frequently selected features of each set of neuroanatomical metric and fused multimodal measures. Training set: 98 patients with FEP and 106 matched HC. Test set: 54 patients with FEP and 48 HC using imaging data acquired on a different magnetic resonance imaging scanner. Using the most frequently selected features from fused structural and diffusion tensor imaging metrics, a classification accuracy of 75.05% was achieved, which was higher than accuracy derived from a single imaging metric. In the independent cohort, average accuracy was 76.54%, derived from combined features selected from cortical thickness, gyrification, fractional anisotropy and mean diffusivity.	Gradient Boosting Decision Tree
Deng *et al.*	2019	A Random Forest algorithm was applied to tractography-based diffusion properties obtained to investigate the machine-learning discriminative power of WM disconnectivity. The classifier was trained by 80% of the sample. In validation using the held-up 20% of the sample, FEP were distinguished from HC with an overall accuracy of 76.0%. Higher predicted probability scores were found in younger patients.	Random Forest

Table 1: Summary of work relating to the detection of SZ using data from structural MRI scans via various artificial intelligence techniques and machine learning algorithms.

Table 2

Study	Year	Methodology	AI/ML
Jafri and Calhoun	2006	In each trial, 2 random patients and 2 random controls were selected as test data, while the rest of the dataset was used for training. 120 trials were performed on the same Neural Network, selecting 65 subjects randomly every time for retraining and 4 remaining subjects for testing to find accuracy of Neural network prediction. The overall mean accuracy for the 120 trials was 75.6%.	Neural network
Calhoun *et al.*	2008	Combined the temporal lobe and the default modes to discriminate subjects with chronic SZ and HC. Participants consisted of 26 HC and 21 chronic SZ outpatients. Temporal lobe and default mode networks were reliably identified in all participants. Controls were correctly classified 95% of the time and SZ patients 92%.	MVPA
Anderson *et al.*	2010	Single-subject independent component analysis (ICA), decomposing an fMRI scan into a set of statistically independent spatial networks, to extract spatial networks and time courses from each subject that have unique relationship with the other components within that subject. This technique is unique, in that it does not require spatial alignment of the scans across subjects. Instead, the classifications are made solely on the temporal activity taken by the subject's unique independent components.	Multivariate Random Forest
Arribas *et al.*	2010	The model selection algorithm is based on the estimation of the posterior probabilities, and three cross-validation based exhaustive neural network complexity model selection algorithms: called cross-validation with weight decay, the Akaikes information criterion and the minimum description length	Stochastic Gradient Learning based on minimization of Kullback-Leibler divergence
Shen *et al.*	2010	This paper examines whether syndromes of SZ could be decoded by some special spatiotemporal patterns of resting-state functional connectivity. An unsupervised-learning classifier combining low-dimensional embedding and self-organised C-means clustering of fMRI was trained to discriminate SZ patients from HC. The performance of the classifier was tested using a leave-one-out cross-validation strategy.	Low-dimensional embedding and self-organised C-means clustering
Yang *et al.*	2010	fMRI measurements and single NP test results from patients and healthy subjects were used. The method consists of four stages: (1) NP with the most discriminating information between the HC and SZ patients are selected to construct a SVM ensemble (NP-SVM). (2) Voxels in the fMRI map contributing to classification are selected to build another SVM ensemble (Voxel-SVM). (3) Components of fMRI activation obtained with ICA are used to construct a single SVM classifier (ICA-SVMC). (4) The above three models are combined into a single module using a majority voting approach to make a final decision (Combined NP-fMRI). Experimental results show that better classification accuracy was achieved by combining genetic and fMRI data than using either alone, indicating that genetic and brain function representing different, but partially complementary aspects, of schizophrenia etiopathology. The analysis produced the following results NP-SVM: 74%, Voxel-SVM: 82%, ICA-SVM: 83% and NP-fMRI: 87%.	SVM

Continued on next page

Table 2

Study	Year	Methodology	AI/ML
Castro *et al.*	2010	This work proposes the application of recursive feature elimination using a machine learning algorithm based on composite kernels. This framework, which evaluates nonlinear relationships between voxels, analyses whole-brain fMRI data from an auditory task experiment that is segmented into anatomical regions and recursively eliminates the uninformative ones based on their relevance estimates, thus yielding the set of most discriminative brain areas for group classification. The collected data was processed using two analysis methods: the general linear model (GLM) and ICA. GLM spatial maps as well as ICA temporal lobe and default mode component maps were then input to the classifier. The mean test accuracy achieved by using ICA default-mode component data was 90%. A mean classification accuracy of up to 95% estimated with a leave-two-out cross-validation procedure was achieved by doing multi-source data classification.	Composite kernels, Linear and Gaussian SVM, Leave-two-out cross-validation
Costafreda *et al.*	2011	104 participants underwent functional magnetic resonance imaging (fMRI) scans while performing a phonological verbal fluency task. Subjects were 32 patients with *SZ* in remission and 40 HC. Neural responses to verbal fluency were examined in each group, and the diagnostic potential of the pattern of the neural responses was assessed with machine learning analysis. The magnitude of activation in the anterior cingulate, left dorsolateral prefrontal cortex and right putamen was greater in patients with *SZ*.	SVM
Fan *et al.*	2011	A novel algorithm is introduced for discriminant analysis of functional brain networks jointly at an individual level. The functional connectivity patterns of different individuals are analysed on the Grassmann manifold by adopting a principal angle based Riemannian distance. In conjunction with a SVM classifier, a forward component selection technique is proposed to select independent components for constructing the most discriminative functional connectivity pattern.	SVM, Linear kernel, Radial basis function kernel, Sigmoid kernel
Du *et al.*	2012	A novel method is introduced to extract classification features from fMRI data collected at rest or during the performance of a task. By combining a two-level feature identification scheme with kernel PCA and Fishers linear discriminant analysis (FLDA), they achieved high classification rates in discriminating HC from *SZ* patients. Experimental results using leave-one-out cross-validation show that features extracted from the default mode network lead to a classification accuracy of over 90% in both datasets. Also, using a majority vote method that uses multiple features, a classification accuracy of 98% in auditory oddball task and 93% in rest data was achieved. Results also suggest that there may be different advantages to using resting fMRI data or task fMRI data.	Fisher's linear discriminant analysis, Default mode network, Majority vote, Leave-one-out cross-validation
Liu *et al.*	2012	Investigated *SZ* inheritance based on the whole-brain resting-state functional connectivity at the individual subject level. PCA was applied to reduce the dimensionality of the functional connectivity feature space. One-against-one pattern classifications were made amongst three groups (i.e. patients diagnosed with *SZ*, healthy siblings, and HC after preprocessing), resulting in an 80.4% separation between patients with *SZ* and HC, a 77.6% separation between *SZ* patients and their healthy siblings, and a 78.7% separation between healthy siblings and healthy controls, respectively. These results suggest that the healthy siblings of *SZ* patients have an altered resting-state functional connectivity pattern compared with HC. Thus, healthy siblings may have a potential higher risk for developing *SZ* compared with the general population.	Nonlinear SVM with polynomial kernel
Venkataraman *et al.*	2012	Whole brain temporal correlations have been estimated using resting-state fMRI data and free surfer cortical parcellations. A multivariate classification method was then used to indentify brain connections that distinguish *SZ* patients from HC. Relative to HC, *SZ* patients exhibited co-existing patterns of increased connectivity between parietal and frontal regions, and decreased connectivity between parietal and temporal regions, and between the temporal cortices bilaterally. The decreased parieto-temporal connectivity was associated with the severity of patients' positive symptoms, while increased fronto-parietal connectivity was associated with patients' negative and general symptoms.	Multivariate classification
Yoon *et al.*	2012	Evaluated fMRI data from subjects while they completed the AX version of the Continuous Performance Task (AX-CPT). Behavioral data yielded an average classification accuracy of 57.8% for all subjects. The group specific accuracies were 64.7% and 51.0% for controls and patients respectively. Classification of left dorsolateral prefrontal cortex fMRI data resulted in an overall accuracy of 61.8% with 64.7% of healthy subject and 58.8% patients being correctly classified compared to their DSM-IV diagnosis. Classifications based on whole brain network fMRI data resulted in an overall accuracy of 58.8%, with 62.7% of healthy subject and 54.9% patients being correctly classified.	Linear DFA, Leave-one-out cross-validation
Anderson and Cohen	2013	Functional network connectivity (FNC) is a method of analyzing the temporal relationship of anatomical brain components, comparing the synchronicity between patient groups or conditions. The *SZ* patients showed significantly less clustering (transitivity) among components than healthy controls ($p < 0.05$, corrected) with networks less likely to be connected, and also showed lower small-world connectivity than healthy controls. Using only these connectivity measures, a SVM classifier (without parameter tuning) could discriminate between *SZ* patients and HC with 65% accuracy. This implies that the global functional connectivity between resting-state networks is altered in *SZ*, with networks more likely to be disconnected and behave dissimilarly for diseased patients.	SVM
Arbabshirani *et al.*	2013	One session of resting-state fMRI data was collected from 28 healthy and 28 *SZ* patients and the data was divided into separate training set (16 HC + 16 *SZ*) and testing set (12 HC + 12 *SZ*) randomly. Several linear and non-linear classification methods (Linear discriminant classifier, Fisher's linear classifier, logistic linear classifier, linear perception, k-nearest neighbour (KNN), naïve Bayes, quadratic classifier, decision tree, neural networks, SVM) were trained using a training dataset and evaluate with a separate testing dataset.	Various (10 types) linear and nonlinear classifier
Fekete *et al.*	2013	This paper introduces how a classification framework complements complex network analysis (CNA) by providing an efficient and objective means of selecting the best network model characterizing given functional connectivity data. We describe a novel kernel-sum learning approach, block diagonal optimization (BDopt), which can be applied to CNA features to single out graph-theoretic characteristics and anatomical regions of interest underlying discrimination, while mitigating problems of multiple comparisons. BDopt performed best achieving a success rate of 100% with Fishers exact probability test confirming the results to be significant (p = 0.00005).	Complex network analysis, Block diagonal optimization.

Continued on next page

Table 2

Study	Year	Methodology	AI/ML
Yu *et al.*	2013	The objective of this study was to use multiclass pattern analysis to investigate the inheritable characters of *SZ* at the individual level, by comparing whole-brain resting-state functional connectivity of patients with *SZ* to their healthy siblings. A linear SVM along with PCA was used to solve the multi-classification problem. The multi-class pattern analysis achieved 62.0% accuracy. The experimental results revealed indication of differences in functional connectivity patterns in the healthy siblings of *SZ* patients compared to other healthy individuals who have no relations with the patients.	SVM, PCA, Leave-one-out cross-validation
Yu *et al.*	2013	To address the issue of high similarity between major depression and *SZ* behavioral symptoms, this study sought to investigate the whole-brain resting-state fMRI of major depression and *SZ* by using multivariate pattern analysis. A SVM in conjunction with intrinsic discriminant analysis was used to solve the multi-classification problem. This preliminary study suggests that altered connections within or across the default mode network and the cerebellum may account for the common behavioral symptoms between major depression and schizophrenia. In addition, connections associated with the prefrontal cortex and the affective network showed promise as biomarkers for discriminating between the two disorders.	SVM, Intrinsic DA, Leave-one-out cross-validation
Anticevic *et al.*	2014	Using resting-state fMRI, we characterised thalamic connectivity in 90 schizophrenia patients versus 90 matched controls via: (1) Subject-specific anatomically defined thalamic seeds; (2) anatomical and data-driven clustering to assay within-thalamus dysconnectivity; and (3) machine learning to classify diagnostic membership via thalamic seed-based connectivity. Schizophrenia analyses revealed functionally related disturbances: Thalamic over-connectivity with bilateral sensorymotor cortices, which predicted symptoms, but thalamic under-connectivity with prefrontalstriatal-cerebellar regions relative to controls, possibly reflective of sensory gating and top-down control disturbances.	Linear SVM, Leave-one-out cross-validation
Brodersen *et al.*	2014	fMRI dataset of *SZ* patients and HC performing a numerical n-back working-memory task was used to construct a dynamic causal model (DCM) using the generative-embedding approach of a visualparietalprefrontal network to define a model-based feature space for the subsequent application of supervised and unsupervised learning techniques. A linear SVM was able to predict individual diagnostic labels significantly more accurately (78%) from DCM-based effective connectivity estimates than from functional connectivity (62%) or local activity within the same regions (55%). Second, an unsupervised approach based on variational Bayesian Gaussian mixture modelling provided evidence for two clusters which mapped onto patients and controls with nearly the same accuracy (71%) as the supervised approach.	Linear SVM, Variational Bayesian Gaussian mixture
Castro *et al.*	2014	MKL finds an automatic combination of kernel functions that can be applied to multiple data sources. By analyzing this combination of kernels, the most informative data sources can be found, hence providing a better understanding of the analysed learning task. This paper presents a methodology based on a new MKL algorithm (ν-MKL) capable of achieving a tunable sparse selection of features' sets (brain regions' patterns) that improves the classification accuracy rate of healthy controls and *SZ* patients by 5% when phase data is included.	L-norm and Lp-norm MKL
Guo *et al.*	2014	Largest population reported in the literature for a resting-state study. Voxel-based morphometry was also used to investigate GM and WM volume changes. Changes were correlated with illness duration/symptom severity and a SVM analysis assessed predictive validity. A network involving the inferior parietal lobule, superior parietal gyrus, precuneus, superior marginal, and angular gyri was by far the most affected (68% predictive validity compared with 82% using all connections) and different components correlated with illness duration and positive and negative symptom severity.	SVM
Watanabe *et al.*	2014	Introduced a novel efficient optimization algorithm based on the augmented Lagrangian and the classical alternating direction method, which can solve both fused Lasso and GraphNet regularised SVM with little modification. Experiments on simulated data and resting state scans from a large *SZ* dataset show that the proposed approach can identify predictive regions that are spatially contiguous in the 6-D "connectome space", offering an additional layer of interpretability that could provide new insights about various disease processes. the fused Lasso regularised SVM yielded the best classification accuracy at 88.2% using 92 features, followed by 85.6% from GraphNet which used 104 features; Lasso and Elastic-net both achieved 77.0% classification accuracy using 230 and 232 features respectively.	Fused Lasso and GraphNet regularised SVM
Cheng *et al.*	2015	Analysed functional connectivity using the largest resting-state neuroimaging dataset reported to date in the *SZ* literature. An exhaustive brain-wide association study at both regional and voxel-based levels enabled a continuous data-driven discovery of the key aberrant circuits in *SZ*. Results identify the thalamus as the key hub for altered functional networks in patients. Increased thalamus-primary somatosensory cortex connectivity was the most significant aberration in *SZ* patients. Overall, a number of thalamic links with motor and sensory cortical regions showed increased connectivity in *SZ* patients, whereas thalamo-frontal connectivity was weakened.	SVM
Chyzhyk *et al.*	2015	Auditory hallucinations (AH) are a symptom that is most often associated with *SZ*, but patients with other neuropsychiatric conditions, and even a small percentage of healthy individuals, may also experience AH. This paper addresses the problem of classifying 26 *SZ* patients with history of AH, and 14 *SZ* patients without a history of AH, and 28 HC. Testing two kinds of neuroimaging features: (a) functional connectivity measures computed by lattice auto-associative memories (LAAM), and (b) local activity measures, including regional homogeneity (ReHo) and fractional amplitude of low frequency fluctuations (fALFF). Discrimination between patients with and without lifetime AH was highest, while discrimination between *SZ* patients and HC participants was worst, suggesting that classification according to the symptom dimension of AH may be more valid than discrimination on the basis of traditional diagnostic categories. ALFF showed poor discriminative ability. fALFF achieved 100% accuracy, sensitivity, and specificity and performed significantly better than ReHo ($p < 0.01$), which achieved 97% accuracy.	Linear SVM
Kaufmann *et al.*	2015	Using rs-fMRI the standard deviation in blood-oxygen-level-dependent signal amplitude and the functional connectivity across a range of functional brain networks were characterised. A model regularised linear discriminant analysis (shrinkage LDA) classifier was trained on the full set of edgewise correlation coefficients using leave-one-out cross-validation. The strongest differences in connectivity implicated within-sensorimotor and sensorimotor-thalamic connections.	Regularised LDA, Leave-one-out cross-validation

Continued on next page

Table 2

Study	Year	Methodology	AI/ML
Pouyan and Shahamat	2015	To obtain the set of features with large discrimination, a two-sample t-test method is used.	ICA, PCA, Various, Leave-one-out cross-validation
Mikolas *et al.*	2016	Applied Linear kernel SVM to the resting-state functional connectivity (rsFC) within the default mode network, the salience network and the central executive network. The classification accuracy was not significantly affected by medication dose, or by the presence of psychotic symptoms. The functional connectivity within the default mode or the central executive networks did not yield classification accuracies above chance level. Seed-based functional connectivity maps can be utilised for diagnostic classification, even early in the course of *SZ*. These results support the role of the anterior insula/ salience network in the pathophysiology of FEP.	Linear SVM
Peters *et al.*	2016	Subcortical-cerebellar systems are known to be altered in schizophrenia. In particular, intrinsic functional brain connectivity (iFC) between these systems has been consistently demonstrated in patients. While altered connectivity is known for each subcortical-cerebellar system separately, it is unknown whether subcortical-cerebellar systems connectivity patterns with the cortico-thalamic system are comparably altered across systems, i.e. if separate subcortical-cerebellar systems connectivity patterns are consistent across patients. To investigate this question rs-fMRI of *SZ* patients and HC were assessed. Independent component analysis of fMRI data revealed cortical intrinsic brain networks (NW) with time courses representing proxies for cortico-thalamic system activity. Subcortical-cerebellar systems activity was represented by fMRI-based time courses of selected ROI (striatum, MTL, amygdala, cerebellum). Correlation analysis among ROI- and NW-time courses yielded individual connectivity matrices [connectivity between NW and ROI (allROI-NW, separate ROI-NW), only NW (NW-NW), and only ROI (allROI-allROI)] as main outcome measures, which were classified by SVM leave-one-out cross-validation. Differences in classification accuracy were statistically evaluated for consistency across subjects and systems. Correlation matrices based on allROI-NW yielded 91% classification accuracy, which was significantly superior to allROI-allROI and NW-NW (56 and 74%, respectively). Considering separate subcortical-cerebellar systems, cerebellum-NW and MTL-NW reached highest accuracy values with 91 and 85%, respectively, while those of striatum-NW and amygdala-NW were significantly lower with about 65% classification accuracy. Results provide initial evidence for differential consistency of altered intrinsic connectivity patterns between subcortical-cerebellar systems and the cortico-thalamic system.	SVM, Leave-one-out cross-validation
Yang *et al.*	2016	Refer to Table 1	MLDA, SVM
Skaatun *et al.*	2017	Few studies have used a data-driven approach for characterizing pathological interactions between regions in the whole brain and evaluated the generalizability across independent samples. To overcome this issue, this study collected rs-fMRI data from 3 independent samples. A whole-brain data-driven definition of network nodes and regularised partial correlations was used to evaluate and compare putatively direct brain network node interactions between groups. The clinical utility of the functional connectivity features and the generalizability of effects across samples were evaluated by training and testing multivariate classifiers in the independent samples using machine learning, using regularised linear discriminant analysis classifiers on edgewise connectivity. Univariate analyses revealed 14 network edges with consistent reductions in functional connectivity encompassing frontal, somatomotor, visual, auditory, and subcortical brain nodes in patients with *SZ*. This study strongly supports the generalizability of connectivity alterations across different scanners and heterogeneous samples.	Multivariate regularised LDA
Chen *et al.*	2017	A rs-fMRI data-driven approach that included local functional connectivity density (FCD) analysis combined with MVPA was used to compare the three groups. Based on the results of the MVPA, the local FCD value in the orbitofrontal cortex (OFC) can differentiate depression patients from *SZ* patients. Subsequent functional connectivity analysis indicated that the connection in the prefrontal cortex was significantly lower in people with *SZ* compared to people with depression and HC. These results suggested that the resting-state functional connectivity pattern in the prefrontal cortex may be a transdiagnostic difference between depression and *SZ* patients.	Linear SVM, MVPA
Kaufmann *et al.*	2017	To address the outstanding issue of pervasiveness across cognitive tasks between patients with *SZ* and bipolar disorder, a classification framework was designed to estimate functional network connectivity from fMRI data obtained during five cognitive tasks (0-back, 2-back, go/no-go, recognition of positive faces, negative faces). An additional 1615 fMRI datasets was also used. Each group was tested for main effects of task and their interactions, and used machine learning to classify task labels and predict cognitive domain scores from brain connectivity. Importantly, there was evidence that brain network dysfunction in severe mental disorders is not confined to specific cognitive tasks and show that the connectivity backbone common to all tasks is predictive of cognitive domain traits.	5-class regularised LDA, k-fold cross-validation model
Guo *et al.*	2017	28 *SZ* patients, 28 family-based controls (FBC) and 40 HC underwent rs-fMRI. Voxel-mirrored homotopic connectivity (VMHC), receiver operating characteristic (ROC) curve and SVM were used to process the data. ROC analysis exhibited that the VMHC values in these brain regions might not be ideal biomarkers to distinguish the patients from the FBC/HC. However, SVM analysis indicated that a combination of VMHC values in the precuneus and lingual gyrus/cerebellum lobule VI might be used as a potential biomarker to distinguish the patients from the FBC with a sensitivity of 96.43%, a specificity of 89.29%, and an accuracy of 92.86%. Results suggested that patients with *SZ* have decreased homotopic connectivity in the motor and low level sensory processing regions. Neuroimaging studies can adopt family-based case-control design as a viable option to reduce the confounding effects of environmental factors on schizophrenia.	SVM, Receiver operating characteristic (ROC) curve
Iwabuchi and Palaniyappan	2017	Examined specific regional and longer-range abnormalities in sensory and thalamic circuits in *SZ* patients, and whether these patterns are strong enough to discriminate symptomatic patients from HC. Using rs-fMRI data, conjunction maps of regional homogeneity (ReHo) and fractional amplitude of low-frequency fluctuations (fALFF) was derived to inform further seed-based Granger causality analysis (GCA) to study effective connectivity patterns. ReHo, fALFF and GCA maps were entered into a multiple kernel learning classifier, to determine whether patterns of local and effective connectivity can differentiate controls from patients. Visual cortex shows both ReHo and fALFF reductions in patients. Visuothalamic effective connectivity in patients was significantly reduced.	MKL

Continued on next page

Table 2

Study	Year	Methodology	AI/ML
Yang *et al.*	2017	By applying a multi-task classification framework (logistic regression as base model) to large size multi-site SZ rs-fMRI dataset, Yang *et al.* observed that SZ patients had widespread deficits in the brain. The most informative and robustly selected functional connectivity features were between and within functional networks such as the default mode network (DMN), the fronto-parietal control network, the subcortical network, and the cingulo-opercular task control network. This finding validated the dysconnection hypothesis of SZ and shed light on the details of the impaired functional connectivity.	Multi-task classification, 10-fold cross-validation
Bae *et al.*	2018	Global and local parameters of functional connectivity were extracted from fMRI data for classification. Various classifiers were applied: k-nearest neighbor, SVM, decision tree, naïve Bayes, and DA classifiers. Decreased global and local network connectivity in subjects with SZ was observed, particularly in the anterior right cingulate cortex, the superior right temporal region, and the inferior left parietal region as compared to HC.	Various (5 types), 10-fold cross-validation
Li *et al.*	2019	Four classifiers: k-nearest neighbours ($k = 5$); linear SVM, Radial basis kernel SVM and LDA were employed. Connective strengths were estimated by Pearson correlation for each pair of brain regions partitioned according to automated anatomical labelling atlas. Subsequently, consensus connections with high discriminative power were extracted under the circumstance of the best classification accuracy. In general, all combinations of feature selection methods and classification methods accomplished comparable performance in distinguishing patients with SZ from HC.	KNN, Liner SVM, Radial basisSVM, LDA
Chatterjee *et al.*	2019	In this study, we aim to identify the brain regions, potentially responsible for the working memory dysfunction, using fMRI data involving SIRP task. Classification guided feature selection is done using SVM and 1-nearest neighbor (1-NN) classifiers. This study reveals several brain regions like cerebellum, inferior temporal gyrus, superior temporal gyrus, superior frontal gyrus, insula, and amygdala that have been reported in the existing literature, thus validating the proposed approach. Other functional changes were also identifiedin the brain regions, such as Heschl gyrus and the vermian area, which have not been reported in the literature involving working memory studies amongst schizophrenia patients.	SVM, k-nearest neighbours
Kalmady *et al.*	2019	An ensemble model—EMPaSchiz (read as Emphasis; standing for Ensemble algorithm with Multiple Parcellations for Schizophrenia prediction) was introduced. The model stacks predictions from several single-source models (SSM), each based on features of regional activity and functional connectivity, over a range of different a priori parcellation schemes. First, EMPaSchiz trained 84 different L2-regularised logistic regression classifiers, each being treated as a SSM. It then trained a single L2-regularised logistic regression model to take the prediction probabilities computed by each SSM, this is a multi-source model (MSM). EMPaSchiz is first to be reported that has been trained and validated exclusively on data from drug-naive patients diagnosed with SZ.	L2-regularised Logistic regression

Table 2: Summary of work relating to the detection of SZ using data from functional MRI scans via various artificial intelligence techniques and machine learning algorithms.

Table 3

Study	Year	Methodology	AI/ML
Caan *et al.*	2006	A technique called 'shaving' is introduced to automatically extract the combination of relevant image regions in a comparative study. A study into schizophrenia using diffusion tensor imaging (DTI) serves as an application. Conventional voxel-based analysis found a decreased fractional anisotropy (FA) in a part of the genu of the corpus callosum and an increased FA in larger parts of white matter. The proposed method reproduced the decrease in FA in the corpus callosum and found an increase in the posterior limb of the internal capsule and uncinate fasciculus. A correlation between the decrease in the corpus callosum and the increase in the uncinate fasciculus was demonstrated.	LDA, PCA
Caprihan *et al.*	2008	Discriminant Principal component analysis (DCPA) was applied to DT-based fractional anisotropy images to distinguish age-matched SZ subjects from HC. The performance of the proposed method was evaluated by the one-leave-out method. For this fractional anisotropy dataset, the classification error with 60 components was close to the minimum error and that the Mahalanobis distance was twice as large with DPCA, than with PCA. Finally, by masking the discriminant function with the white matter tracts of the Johns Hopkins University atlas, the left superior longitudinal fasciculus as the tract which gave the least classification error. In addition, with six optimally chosen tracts the classification error was zero.	DPCA
Ingalhalikar *et al.*	2010	Using anatomically meaningful features computed from the DTI data, a nonlinear SVM pattern classifier was trained. The method begins with high dimensional elastic registration of DT images followed by a feature extraction step that involves creating a feature by concatenating average anisotropy and diffusivity values in anatomically meaningful regions. Feature selection is performed via a mutual information based technique followed by sequential elimination of the features. The classifier assigns each test subject with a probabilistic abnormality score that indicates the extent of pathology. The abnormality scores clearly separate the groups and the high classification accuracy indicates the prospect of using the scores as a diagnostic and prognostic marker.	Nonlinear SVM
Rathi *et al.*	2010	Two different models of the dMRI data, namely, spherical harmonics (SH) and the two-tensor model (F2T) was used to classify FEP. The algorithm works by first computing several diffusion measures from each model. An affine-invariant representation of each subject is then computed, thus avoiding the need for registration. This representation is used within a kernel based feature selection algorithm to determine the biomarkers that are statistically different between the two populations.	K-nearest neighbours, Parzen window classifier, SVM
Ardekani *et al.*	2011	The objective of this research was to determine whether fractional anisotropy (FA) and mean diffusivity (MD) maps derived from DTI of the brain are able to reliably differentiate patients with schizophrenia from healthy volunteers. FA and MD maps were estimated from the DTI data and spatially normalised to the Montreal Neurologic Institute standard stereotactic space. Patterns of water self-diffusion in the brain as estimated by DTI can be used in conjunction with automated pattern recognition algorithms to reliably distinguish between patients with schizophrenia and normal control subjects.	Fisher's LDA

Continued on next page

Table 3

Study	Year	Methodology	AI/ML
Squarcina *et al.*	2015	This work investigated dynamic susceptibility contrast magnetic resonance imaging, and cerebral blood flow and volume, along with mean transit time behavior in the whole brain and in selected regions of interest, in particular the left and right frontal, parietal, temporal and occipital lobes, insula, caudate and cerebellum. The distribution of values of perfusion indexes were used as features in a SVM classifier. Mean values of blood flow and volume were slightly lower in patients, and the difference reached statistical significance in the right caudate, left and right frontal lobes, and in left cerebellum. Linear SVM reached an accuracy of 83% in the classification of patients and HC, with the highest accuracy associated with the right frontal lobe and left parietal lobe.	SVM

Table 3: Summary of work relating to the detection of *SZ* using data from diffusion-weight MRI, diffusion tensor imaging and perfusion MRI scans via various artificial intelligence techniques and machine learning algorithms.

Table 4

Study	Year	Methodology	AI/ML
Knott *et al.*	1999	This study examined P300s in symptomatically stable, medicated, chronic *SZ* patients and HC performing a visual continuous performance task utilizing degraded stimuli to burden encoding processes. Performance analysis found slower response times, fewer target detections and more false alarms in patients than in HC. Analysis of the ERP showed P300 amplitudes of patients to be significantly smaller than those of controls and, unlike controls, patients failed to exhibit significant target vs. non-target P300 amplitude differences. Three separate DFA and jackknife classification were performed based on target amplitude values, non-target amplitude values and both values entered together. The overall classification rate was high for all three analyses with amplitudes of target (89.3%) and non-target (92.3%) stimuli yielding similar overall classification rates, whereas the use of both stimulus-related amplitudes in the highest classification rate of 100%	DFA, Jackknife classification
Neuhaus *et al.*	2011	Subjects completed the Attention Network Test while an EEG was recorded. Target-locked N1 and P3 ERP components were constructed and submitted to different classification analyses without a priori hypotheses. Standardised source localization was applied to estimate neural sources of N1 and P3 deficits in schizophrenia. Nine different classification methods were applied. The classification accuracy was obtained using only very few ERP components.	SVM (linear, quadratic and radial basis kernels), LDA, Quadratic discriminant analysis (QDA), KNN, Naïve Bayes with equal and unequal variances and Mahalanobis classification.
Iyer *et al.*	2012	Responses were obtained in a paired-stimulus paradigm, in which an auditory stimulus S1 is followed by an identical S2. The amplitude and latency of the P50 and N100 components in response to the S1 and S2 stimuli were measured in each single trial and used as features to classify the responses into two groups. The Random Forest classifier was used to develop classification rules. The S1 latency was the most significant discriminatory feature ($p < 0.01$) followed by S2 latency ($p < 0.01$). The S2 amplitude, though relatively larger in normal subjects ($p < 0.05$), was the least discriminatory feature.	Random Forest, 10-fold stratified cross-validation
Laton *et al.*	2014	Combination of features extracted from the auditory and visual P300 paradigms and the mismatch negativity paradigm. The EEG-data were high- and low-pass filtered, epoched and averaged. Features (latencies and amplitudes of component peaks) were extracted from the averaged signals. The resulting dataset was used to train and test classification algorithms, first on separate paradigms and then on all combinations. For at least two classifiers the performance increased significantly by combining paradigms compared to single paradigms. The classification accuracy increased from at best 79.8% when trained on features from single paradigms, to 84.7% when trained on features from all three paradigms.	Naïve Bayes, SVM and decision tree, with two of its improvements: adaboost and Random Forest
Neuhaus *et al.*	2014	Investigated subjects with an auditory click-conditioning/oddball paradigm. P50 and N1 gating ratios as well as target-locked N1 and P3 components were submitted to conventional general linear models and to explorative machine learning algorithms. Repeated measures ANOVAs revealed significant between-group differences for the oddball-locked N1 and P3 components but not for any gating measure. Machine learning-assisted analysis achieved 77.7% balanced classification accuracy using a combination of target-locked N1 and P3 amplitudes as classifiers. Fifteen different classification methods were applied.	LDA and QDA (with their diagonal variants), SVM (linear, polynomial, radial basis and multilayer perceptron kernels), Naïve Bayes, KNN (Euclidean and cosine distance measures) and Mahalanobis classification
Johannesen *et al.*	2016	Subjects completed a Sternberg Working Memory Task (SWMT) during EEG recording. The 1-norm SVM method was used to build EEG classifiers of SWMT trial accuracy (correct vs. incorrect; Model 1) and diagnosis (HC vs. *SZ*; Model 2). Model 1 correctly classified trial accuracy at 84% in HC, and at 74% in *SZ* data. Model 2 identified frontal theta at baseline and frontal alpha during retrieval as primary classifiers of diagnosis, providing 87% classification accuracy as a discriminant function.	1-norm SVM

Continued on next page

Table 4

Study	Year	Methodology	AI/ML
Shim *et al.*	2016	In this study, both sensor-level and source-level features extracted from EEG signals recorded during an auditory oddball task were used for the classification. The results demonstrated higher classification accuracy when source-level features were used together with sensor-level features, compared to when only sensor-level features were used. In addition, the selected sensor-level features were mostly found in the frontal area, and the selected source-level features were mostly extracted from the temporal area, which coincide well with the well-known pathological region of cognitive processing in *SZ* patients.	SVM, Leave-one-out cross-validation
Taylor *et al.*	2017	EEG data were acquired from subjects whilst they listened to three auditory oddball paradigms comprising sequences of tones which deviated in 10% of trials from regularly occurring standard tones. Using SVM and Gaussian processes classifiers, a balanced accuracy of up to 80.48% was achieved.	SVM, Gaussian processes classifiers, MVPA
Krishnan *et al.*	2020	Multivariate Empirical Mode Decomposition (MEMD) is used to decompose the EEG signal into Intrinsic Mode Functions (IMF) signal. The randomness measure of the IMF signal is determined by computing the entropy of the signal. Various entropy measures were measured from the IMF signal and ML techniques were used to discriminate patients from HC.	Various, SVM (Radial Basis Function)

Table 4: Summary of work relating to the detection of *SZ* using data from electroencephalogram scans via various artificial intelligence techniques and machine learning algorithms.
